# Antioxidant, Anti-Inflammatory, Antimicrobial, and Anticancer Activities of Pomegranate Juice Concentrate

**DOI:** 10.3390/nu15122709

**Published:** 2023-06-11

**Authors:** Hosam M. Habib, Hamada El-Gendi, Esmail M. El-Fakharany, Mohamed G. El-Ziney, Ahmed F. El-Yazbi, Fatima T. Al Meqbaali, Wissam H. Ibrahim

**Affiliations:** 1 Research & Innovation Hub, Alamein International University (AIU), Alamein City 5060310, Egypt; hhabib@aiu.edu.eg (H.M.H.); ahmed.fawzy.aly@alexu.edu.eg (A.F.E.-Y.); 2 Bioprocess Development Department, Genetic Engineering and Biotechnology Research Institute, City of Scientific Research and Technological Applications (SRTA City), New Borg El Arab P.O. Box 21934, Egypt; elgendi1981@yahoo.com; 3 Protein Research Department, Genetic Engineering and Biotechnology Research Institute GEBRI, City of Scientific Research and Technological Applications (SRTA City), New Borg El Arab P.O. Box 21934, Egypt; esmailelfakharany@yahoo.co.uk; 4 Dairy Science and Technology Department, Faculty of Agriculture, Alexandria University, Alexandria P.O. Box 21545, Egypt; elziney@yahoo.com; 5 Department of Pharmacology and Toxicology, Faculty of Pharmacy, Alexandria University, Alexandria 21521, Egypt; 6 Faculty of Pharmacy, Alamein International University (AIU), Alamein City 5060310, Egypt; 7 Department of Nutrition and Health, College of Medicine and Health Sciences, United Arab Emirates University, Al Ain P.O. Box 15551, United Arab Emirates; fatmadiab@uaeu.ac.ae

**Keywords:** pomegranate juice concentrate, functional food, DNA and protein damage, enzyme inhibition, antibacterial, anticancer, nutraceutical

## Abstract

Pomegranate juice concentrate (PJC) is a rich source of polyphenols, which exhibit significant antioxidant activity and potential health benefits for disease prevention and therapy. In this study, the polyphenolic profile of PJC was investigated for the first time, and it was found that PJC can inhibit oxidative damage to bovine serum albumin (BSA) and deoxyribonucleic acid (DNA), as well as acetylcholinesterase, α-amylase, and tyrosinase activities. The primary polyphenols identified in PJC were 4-Hydroxy-3-Methoxybenzoate, epicatechin, catechin, rutin, ferulic acid, P-coumaric acid, and cinnamic acid. Additionally, PJC demonstrated potent antibacterial effects against human pathogens such as *Streptococcus mutans* and *Aeromonas hydrophila* and dose-dependently reduced the proliferation of colorectal, breast, and hepatic cancer cells via apoptosis. Furthermore, PJC blocked B-cell lymphoma 2 (BCl-2) and the expression of a potent cyclin-dependent kinase inhibitor (P21) and enhanced tumor protein (P53) expression, compared to both untreated cells and cells treated with fluoropyrimidine 5-fluorouracil (5-FU). As a result, PJC may be a beneficial ingredient in the formulation of emerging natural-compound-based chemotherapy and functional foods and could be utilized by the food, nutraceutical, and pharmaceutical industries.

## 1. Introduction

Pomegranate juice concentrate (PJC) is prepared from pomegranate fruit juice, which belongs to the Punicaceae family, Punica genus, and *Punica granatum* L. species [1,2]. Pomegranates are considered an ancient fruit, which can be grown widely in semi-arid land, arid land, and poor soil, with good yields. They can thrive in low moisture and tolerate saline water; therefore, they are broadly spread and cultivated throughout the world [3,4,5].

PJC can be produced traditionally or commercially. Commercial production involves boiling pomegranate juice, filtration, enzyme addition, clarification, and evaporation under vacuum steps [6]. Therefore, these steps produce a thick sour and sweet PJC syrup, which is slightly astringent and somewhat ruby in color [7,8,9]. PJC is commonly used as a flavoring agent, sweetener agent, sauce, and salad dressing. It is also used in dairy products, meat products, and many dishes to improve aroma and taste characteristics [2,5,6,10,11,12,13]. Pomegranates have been extensively used as a source of folk medicine for a long time. Recently, researchers have shown that pomegranates have many potential beneficial health effects, including anti-diabetic, antioxidant, anti-carcinogenic, anti-inflammatory, anti-diarrheal, anti-microbial, and neuroprotective activities [2,3,12,14].

PJC is a rich source of bioactive compounds and nutrients that have been shown to provide potential health benefits [15,16,17]. The regular consumption of pomegranate juice or PJC has been linked to a reduction in blood pressure, improved blood lipid levels, and reduced oxidative stress [18,19]. Additionally, pomegranate juice concentrate is being investigated for its potential effects on gut health, with some studies suggesting that it may promote the growth of beneficial gut bacteria [20,21,22].

In addition, pomegranate juice concentrate has been found to have potential benefits for athletes and physically active individuals as it may help to reduce muscle soreness and oxidative stress when consumed before exercise [23,24,25]. Pomegranate juice concentrate is also used as a functional food ingredient and dietary supplement and is being investigated for its potential applications in the pharmaceutical and cosmetic industries [26,27]. Studies have shown that pomegranate extract, derived from pomegranate juice concentrate, may have antibacterial, antiviral, and anti-inflammatory effects and may promote wound healing and skin health [28,29].

Several investigations have been carried out regarding the physicochemical and biochemical effects, total flavonoid and phenolic content, antioxidant activities, and biological activities of PJC [2,3,12,14]. Nevertheless, as far as we know, several of the bioactivities of PJC investigated in the current study have not yet been reported. Hence, the primary purpose of the present investigation was to account for some unreported health-promoting activities of PJC associated with bioactive compounds, protein, DNA damage and inhibiting important clinical enzymes including Acetylcholinesterase, α-amylase, and Tyrosinase, the inhibition of which has been confirmed ot have positive effects on several disease states. Moreover, PJC’s antibacterial effects against nine human pathogens were investigated. Finally, the influence of PJC on apoptosis and the propagation of colon, breast, and hepatic cancer cells was also considered.

## 2. Materials and Methods

### 2.1. Materials

All chemicals utilized in this study were of analytical grade. TPTZ (2,4,6-tri(2-pyridyl)-s-triazine), naphthyl ethylene, DPPH (1,1-diphenyl-2-picrylhydrazyl), diamine dihydrochloride, sodium nitroprusside, sulfuric acid, ammonium molybdate, sodium phosphate, H_2_O_2_, FeSO_4_, 4-hydroxy-3-methoxy benzoic acid, gallic acid, p-coumaric acid, ferulic acid, syringic acid, cinnamic acid, epicatechin, catechin, MTT [3-(4,5-dimethylthiazol-2-yl)-2,5 diphenyltetrazolium bromide], paraformaldehyde, ethidium bromide/acridine orange (EB/AO), RNase A, agarose, trypsin/EDTA, PI dye, and DMSO were acquired from Sigma (St. Louis, MO, USA). Folin–Ciocalteu’s phenol reagent, FeSO_4_–H_2_O, HCl, FeCl_3_, and pBR322 DNA were sourced from Biolabs (Ipswich, MA, USA). DMEM and RPMI-1640 medium were procured from Lonza (Houston, TX, USA). Caco-2 (colon carcinoma), MDA (breast carcinoma), HepG-2 (hepatoma), and HSF (normal somatic cells) cell lines, Streptococcus mutans (ATCC 25175), Staphylococcus aureus (ATCC 25923), Salmonella typhimurium (ATCC 14028), Escherichia coli (ATCC 25922), Pseudomonas aeruginosa (ATCC 27853), Pseudomonas fluorescens (DSM 50090), Aeromonas hydrophila (NNRL 914), Klebsiella pneumoniae (ATCC 13883), and Candida albicans (ATCC 10231) were obtained from ATCC (American Type Culture Collection) (Manassas, VA, USA). Gene JET RNA purification kit was procured from Thermo Scientific (Waltham, MA, USA). In 2022, PJC samples (*n* = 10) were collected from various locations and at different times from the local market.

#### 2.1.1. Sampling

Each sample was diluted with deionized water by a factor of 10 and stored at −20 °C until analysis. Prior to conducting the analyses, the PJC samples were thawed at room temperature.

#### 2.1.2. Sample Extraction

Polyphenolic compounds were obtained from PJC samples using accelerated solvent extraction (ASE) (ASE, 350, Dionex Co., Thermo, Scientific, California, CA, USA) in 11 mL stainless steel extraction cells. Collection was conducted in 40 mL amber vials. The extraction parameters included pressure at 1500 psi; temperature at 25 °C; static time and cycles at 5 and 4 min, respectively; purging for 90 s; and flushing at 75%. Acidified water (pH 2 with HCl) was used for the extraction process [30].

### 2.2. Methods

#### 2.2.1. Total Phenolics (TP)

The total phenolic content in PJC was evaluated using Folin–Ciocalteu’s phenol reagent using spectrophotometric analysis, as described in earlier studies [31]. A standard curve of gallic acid (0–100 mg mL^−1^) was employed for comparison, and the total phenolic content was expressed as mg of gallic acid equivalent (GAE) per 100 g (mg GAE/100 g) of PJC.

#### 2.2.2. Total Flavonoids (TF)

The total flavonoid content of PJC was estimated using a previously described method [31]. For this, a 250 μL solution of each standard or extract was mixed with 75 μL of 5% NaNO_2_ solution and 1.25 mL of H2O. After 6 min, 150 μL of a 10% AlCl_3_ solution was added, followed by 0.5 mL of 1 M NaOH solution after 5 min. The total volume was adjusted to 2.5 mL with H_2_O, and the absorbance of the sample against the blank was measured at 510 nm wavelength. A standard curve of rutin (0–100 mg mL^−1^) was used to determine the total flavonoid content, which was expressed as mg rutin equivalent (RE) per 100 g (mg RE/100 g) of PJC.

#### 2.2.3. Quantification of Phenolic Acids and Flavonoids by HPLC

The HPLC method used in this study was previously described in [30]. The HPLC system consisted of a 1525 Binary HPLC pump (Waters, Milford, MA, USA), a separation module equipped with a 2487 dual UV detector (Waters, Milford, MA, USA), and a 717 plus auto-sampler (Waters, Milford, MA, USA) controlled by Breeze software (version 1.15). Separation was achieved using Waters Xterra RP 18.5 µm 4.6 × 150 mm column. HPLC-grade solvents were filtered through a 0.45 µm filter, and elution was performed using a linear gradient of solvent A (1% acetic acid) and solvent B (acetonitrile) starting with 5% solvent B at 0 min, reaching 7% at 5 min, 9% at 10 min, 12% at 15 min, 15% at 18 min, 16% at 20 min, 18% at 25 min, 20% at 30 min, 22% at 32 min, 25% at 35 min, 28% at 38 min, 30% at 40 min, 31% at 42 min, 32% at 45 min, 34% at 48 min, 35% at 50 min, 40% at 55 min, 50% at 60 min, 95% at 80 min, and 5% at 90 min, followed by a post-run for 5 min. A flow rate of 0.7 mL/min and an injected volume of 20 µL were used. The UV spectra were recorded at 280 nm and 330 nm.

#### 2.2.4. Antioxidant Activity

##### DPPH^•^ Free Radical Scavenging Assay

The scavenging activity of PJC (100 mg/mL), vitamin C (1 mg/mL), and rutin (1 mg/mL) as positive controls was evaluated by measuring their ability to scavenge the 1,1-diphenyl-2-picrylhydrazyl (DPPH^•^) free radicals using a method described previously [32]. The results were expressed as the percentage of inhibition of the DPPH^•^ radical using the following equation:

% Inhibition = (Abs control − Abs sample)/Abs control × 100(1)

where Abs control is the absorbance of the DPPH^•^ solution without the tested sample.

##### ABTS^•^ Free Radical Scavenging Assay

The ability of PJC (100 mg/mL), vitamin C (1 mg/mL), and rutin (1 mg/mL) as positive controls to scavenge the ABTS^•^ free radicals was evaluated using a method described previously [33]. The percentage of inhibition of the ABTS^•^ radical was calculated using Equation (1) as described above.

##### Nitric Oxide Radical Scavenging Analysis

The inhibitory effect of PJC (100 mg/mL), vitamin C (1 mg/mL), and rutin (1 mg/mL) as positive controls on the nitric oxide radical was evaluated using the Griess reaction. A reaction mixture (3 mL) containing 10 mM sodium nitroprusside (2 mL) and 0.5 mL of PJC (100 mg/mL) in phosphate-buffered saline was incubated at 25 °C for 150 min, along with rutin (1 mg/mL) or vitamin C (1 mg/mL) as positive controls. After incubation, 0.5 mL of the reaction mixture was mixed with 1 mL of sulfanilic acid reagent (0.33% in 20% glacial acetic acid) and allowed to stand for 5 min. Finally, 1 mL of 0.1% naphthyl ethylene diamine dihydrochloride was added, mixed, and incubated for 30 min at 25 °C. The nitrite concentration was measured at 540 nm using a method described previously [34]. The percentage inhibition of nitric oxide was calculated using Equation (1) as described above.

##### Ferric Reducing/Antioxidant Power (FRAP) Assay

The ferric reducing/antioxidant power (FRAP) assay was used to evaluate PJC (100 mg/mL), vitamin C (1 mg/mL), and rutin (1 mg/mL) as positive controls, following a previously described method [35]. The FRAP reagent consisted of a mixture of 10 mM TPTZ solution dissolved in 40 mM HCl, 20 mM FeCl_3_ solution, and acetate buffer (0.3 M, pH 3.6) in the ratio of 1:1:10 (*v*/*v*/*v*). Each diluted solution of the tested sample (1 mL) was mixed with freshly prepared FRAP reagent (2 mL), and the reaction mixtures were incubated for 30 min at 37 °C. The absorbance was measured at 593 nm against distilled water as a blank. Standard solutions of ferrous sulfate (0–100 µM) were used for calibration. The FRAP values were expressed as µ mol of Fe (II) and recorded.

##### Total Antioxidant Activity (TAC)

A previously reported method [30] was used to investigate the total antioxidant activity of PJC (100 mg/mL), vitamin C (1 mg/mL), and rutin (1 mg/mL) as positive controls. Briefly, samples with varying concentrations (0.1 mL) were mixed with a reagent solution consisting of 28 mM sodium phosphate, 0.6 M sulphuric acid, and 4 mM ammonium molybdate solutions (0.3 mL). The tubes were covered, and the reaction mixtures were incubated at 95 °C for 90 min. After cooling, the absorbance of the mixture was measured against a blank sample at 695 nm. The blank contained the reagent solution and the solvent. The result was expressed as the absorbance of the sample, in which higher absorbance values indicated greater antioxidant activity.

##### Damage to DNA Caused by Free Radicals

A previously described method [35] was used to evaluate DNA damage. In summary, pBR322 DNA (0.2 μg) in 2 μL of 50 mM PBS with a pH of 7.4 was mixed with samples (4 μL) in a 20 μL Eppendorf tube containing PBS buffer (6 μL). Then, 30% H_2_O_2_ (6 μL) was added, and the reactions were initiated by UV irradiation for 5 min on the surface of a UV transilluminator TFM-26 (UVP, Upland, CA, USA) at a 25 W cm^−2^ intensity and 312 nm wavelength at room temperature. After the reaction, the samples were run on agarose (0.8%), and the gel was stained with ethidium bromide and photographed. The images were analyzed using software Image Lab 4.1 (version 6.1.0 build 7, Bio-Rad, Hercules, CA, USA). VC and rutin were operated as positive controls.

##### The Oxidation of Proteins Caused by AAPH

In the study, bovine serum albumin (BSA) was subjected to oxidation by alkyl peroxyl radical [31]. AAPH (20 mM) was incubated with BSA (0.5 mg/mL) in the presence or absence of different extracts in a shaking water bath for 30 min at 37 °C. A protein sample without AAPH was used as a control. Following oxidation, the protein samples were mixed with loading buffer and heated at 100 °C for 5 min. The samples were then loaded using 10% SDS-PAGE under reducing conditions. The stained gels were imaged using a gel documentation system (ChemiDoc MV, Bio-Rad, Hercules, CA, USA), and the intensity of the protein damage band was determined using software Image Lab 4.1 (version 6.1.0 build 7, Bio-Rad, Hercules, CA, USA). The optical density of each band was standardized and evaluated against the control group. Positive controls, VC, and rutin were also used in the study.

#### 2.2.5. Enzyme Inhibition Activity

##### Assay for Inhibiting Porcine α-Amylase

The porcine α-amylase inhibition activity of PJC, rutin, vitamin C, and acarbose was measured using a previously described method [36]. Briefly, a mixture of 50 µL of each of the test compounds (100 mg/mL PJC, 1 mg/mL rutin, 1 mg/mL vitamin C, or 1 mg/mL acarbose), which were used as positive controls; 50 µL of 0.02 M sodium phosphate buffer (pH 6.9) with 6 mM sodium chloride; and 13 units/mL of α-amylase solution was incubated at 25 °C for 10 min. Then, 50 µL of 1% starch solution in 20 mM sodium phosphate buffer (pH 6.9) with 6 mM sodium chloride was added, and the mixture was incubated at 25 °C for an additional 10 min. Next, 1 mL of dinitrosalicylic acid (DNS) color reagent was added to stop the reaction. The mixture was then heated at 100 °C in a water bath for 10 min and cooled to 25 °C. Finally, 1 mL of deionized water was added, and the absorbance was measured at 540 nm using a 96-well microplate reader. The inhibition of porcine α-amylase was calculated using Equation (1) as described above.

##### Assay for Inhibiting Tyrosinase

A previously described method [36] was used to evaluate the tyrosinase inhibition activity. A L-tyrosine solution (0.5 mg/mL, 4 mL) was dissolved in phosphate buffer (20 mM at pH 6.8) and mixed with kojic acid (1 mg/mL), rutin (1 mg/mL), and vitamin C (1 mg/mL) as positive controls or PJC (100 mg/mL). After adding mushroom tyrosinase (50 units/mL) dissolved in phosphate buffer (0.2 M, pH 6.8), the mixture was incubated for 10 min at 37 °C. The absorbance was measured at 475 nm after 10 min. Deionized water (1 mL) was used as control, and a 50% ethanol solution was used as a blank. The percentage of tyrosinase inhibitory activity was calculated using Equation (1) as described earlier.

##### Assay for Inhibiting Acetylcholinesterase (AChE)

A previously described method [36] was utilized to evaluate the Anti-AChE inhibition activity. To summarize, 100 µL of PJC (100 mg/mL), vitamin C (1 mg/mL), galantamine (1 mg/mL), or rutin (1 mg/mL) as positive controls were added to 325 µL of Tris-HCl buffer (0.05 M, pH 8) and then incubated with 25 µL of anti-acetylcholinesterase (0.28 U/mL) for 15 min. Subsequently, 75 µL of acetylcholine iodide (AChI) solution of 15 mM and 475 µL of 3 mM DTNB solution were added and incubated for 30 min at 25 °C. The absorbance was then measured at 405 nm. The AChE inhibition activity was calculated using Equation (1), as described earlier.

#### 2.2.6. Antimicrobial Activity

##### Antimicrobial Assays

The PJC antimicrobial activity was evaluated against 9 human pathogens using the agar well diffusion method according to the Kirby–Bauer protocol with some modifications [37]. Nine pathogens were included in the study, comprising two Gram-positive bacteria, *Staphylococcus aureus* (ATCC 25923) and *Streptococcus mutans* (ATCC 25175), and six Gram-negative bacteria. including *Escherichia coli* (ATCC 25922), *Pseudomonas aeruginosa* (ATCC 27853), *Salmonella typhimurium* (ATCC 14028), *Aeromonas hydrophila* (NNRL 914), *Klebsiella pneumoniae* (ATCC 13883), and *Pseudomonas fluorescens* (DSM 50090)), in addition to *Candida albicans* (ATCC 10231) as unicellular fungi. The PJC antimicrobial activity was evaluated at four different concentrations, including 175, 350, 525, and 700 µg/well. The pre-inoculation cultures were prepared via separate cultivation of the nine pathogens in nutrient broth (NB) media at 37 °C for 24 h. The agar well diffusion method was performed using four wells aseptically created in a Petri plate of nutrient agar medium inoculated with 100 μL from overnight culture (0.5 McFarland) of one of the nine applied pathogens and incubated at 37 °C for 24 h.

##### Minimum Inhibitory Concentrations (MIC) Assay

Based upon the results of the antimicrobial activity, the minimum inhibitory concentrations (MIC) of PJC were determined for the highest susceptible pathogens (7 pathogens) via a microdilution assay using a 96-well cell culture plate. The pathogens included were *Streptococcus mutans* (ATCC 25175), *Staphylococcus aureus* (ATCC 25923), *Salmonella typhi* (ATCC 14028), *Escherichia coli* (ATCC 25922), *Pseudomonas aeruginosa* (ATCC 27853), and *Pseudomonas fluorescens* (DSM 50090). From overnight culture, 100 μL of the five tested organisms (10^6^ CFU/mL) was injected separately into 96-well cell culture plates along with serial dilution of PJC (35–560 µg). The volume of the mixture was adjusted to 200 µL/well, and the plates were incubated at 37 °C for 24 h. The absorbance was measured at 600 nm using a microplate reader. The results were presented as the minimum inhibitory concentration (MIC), which represents the lowest concentration (in µg/mL) that prevented visible growth of cells.

#### 2.2.7. Anticancer Activity

##### Cytotoxicity

To evaluate the cytotoxicity of PJC against normal human skin fibroblast (HSF) cells, the MTT assay was conducted as previously described [31]. HSF cells were cultured at concentrations of 5.0 × 10^3^ cells per well in a supplemented DMEM medium (Lonza, Walkersville, MD, USA) with 10% fetal bovine serum (FBS) using a sterile 96-well microplate. After overnight incubation, the cells were exposed to PJC at various concentrations of 0.0, 40, 80, 120, 160, 200, and 240 μg/mL and incubated at 37 °C for 24 and 48 h in a 5% CO_2_ incubator. The cells were washed 3 times with PBS to remove dead cells and debris. Then, MTT (200 μL) 0.5 mg/mL was added to specific wells and incubated for a further 3–5 h at 37 °C in a 5% CO_2_ incubator. Then, MTT solutions were removed, and 200 μL of 100 % DMSO was added to dissolve the formed formazan crystals. The absorbance of each well was measured at 570 nm using a microplate reader (BMG LabTech, Ortenberg, Germany), and the values of IC_50_ (half maximal inhibitory concentration) and EC_100_ (safe dose) of PJC were calculated using Graph Pad Prism software (version 6.0).

##### MTT Assay for Cell Proliferation

The PJC anticancer effect was established in vitro using MDA (breast carcinoma), Caco-2 (colon carcinoma), and HepG-2 (hepatoma) cell lines, which were seeded into 3 microplates and incubated until attachment. Caco-2 and MDA cells were cultured and maintained in a DMEM medium supplemented with FBS, 10%, whereas HepG-2 cells were cultured and maintained in a supplemented RPMI-1640 medium with FBS, 10%. The cells were treated with PJC at different concentrations of 40, 80, 120, 160, 200, and 240 µg/mL and incubated for 24 and 48 h at 37 °C under 5% CO_2_ incubator. The anticancer effect of the PJC against the established cancer cell lines was assessed by the MTT method, as mentioned previously. The IC_50_ and EC_100_ values of PJC were measured by the GraphPad Prism 7.0, and the selectivity index (SI) value that was demarcated as the ratio of the IC_50_ on normal HSF cells against the IC_50_ value of each cancer cell line was assessed [38]. The effect of PJC on the morphology of Caco-2, HepG-2, and MDA cells was investigated using inverted phase-contrast microscopy (Olympus, Hamburg, Germany) at concentrations of 40, 80, and 160 μg/mL to obtain associations with untreated healthy cells.

##### Apoptotic Effect Analysis

The apoptotic consequence of PJC on HepG-2 cells was examined by ethidium bromide/acridine orange (EB/AO) and propidium iodide (PI) dye. Fluorescent nuclear staining techniques are as follows: The attached HepG-2 cells were exposed to PJC at 40, 80, and 160 μg/mL for 48 h in a 5% CO_2_ incubator. Subsequently washing the cells 3 times with PBS, cells were fixed with paraformaldehyde 4% and stained with EB/AO or PI (10 µg/mL) (100 µg/mL to separately dye) for 20 min. The nuclear staining was visualized and captured under an inverted fluorescence phase-contrast microscope (Olympus, Tokyo, Japan) with a dichromatic mirror cut-on 505 nm and an excitation filter (480/30 nm). The untouched cells were involved as negative control cells.

##### Analysis of Cell Cycle

The cell cycle analysis for treated HepG-2 cells was examined by flow cytometry in contrast with untreated cells [38]. The attached HepG-2 cells were treated with PJC at 40, 80, and 160 μg/mL for 48 h in a CO_2_ 5% incubator. After washing the cells, HepG-2 cells were detached, resuspended in cold PBS, and fixed with cold ethanol 70% under a mild vortex. The fixed cells were incubated at room temperature for 1 h in PBS containing 5 μg/mL RNase A and stained with PI (Sigma-Aldrich (St. Louis, MO, USA) at an ultimate concentration of 1 mg/mL in deionized water in dark conditions. The cell cycle distribution of the treated HepG-2 cells was estimated on FACS (Partec, Jettingen-Scheppach, Germany) using Mod Fit (version 4.0) and Cell Quist (version 3.2) software at 488 nm. Untreated healthy HepG-2 cells were involved as a reference.

##### Quantitative of Oncogenes Expression

To evaluate the expression of some oncogenes after treatment with PJC, the relative change in p53, Bcl2, p21, and VEGF gene expressions in the preserved cancer cells relative to untouched cancer cells was investigated using the qPCR technique. In brief, total RNAs of treated Caco-2, HepG-2, and MDA cells were extracted at IC50 values for 48 h by JET RNA Gene purification kit (Thermo Scientific, Waltham, MA, USA). The cDNA was synthesized, and qPCR was performed employing specific primers (Forward/Reverse) for each gene and SYBR green kit. The used primers are 5′- TCCGATCAGGAAGGCTAGAGTT-3′/5′-TCGGTCTCCTAA-AAGCAGGC-3′ for Bcl2, 5′-ATGTTTTGCCAACTGGCCAAG-3′/5′-TGAGCAGCGCT-CATGGTG-3′ for p53, 5′-CATATGCGGCTGCTGTTCTA-3′/5′-CCGAAAGCCGTTTCTTGTAG-3′ for β-catenin, and 5′-GGCTTTACTGCTGTACCTCC-3′/5′-CAAATGCTTTCTCCGCTCT-3′ for VEGF. Untreated control cells were included as reference cells. The change in each gene expression in the treated cancer cells, relative to reference cells, was estimated by the equation of 2−∆∆CT (2ˆ(−delta of the threshold cycles (CTs)).

#### 2.2.8. Statistical Analysis

The analytical measurements were conducted in triplicates, and statistical analyses were performed using SPSS for Windows (version 25; SPSS Inc., Chicago, IL, USA). To determine the differences in mean values among the sample varieties, a one-way analysis of variance (ANOVA) was performed, and statistical significance was set at *p* < 0.05. Tukey multiple range tests were used for means separation. The results are presented as mean ± standard deviation values.

## 3. Results and Discussion

### 3.1. Bioactive Compounds

#### 3.1.1. Total Phenolics

The total phenolic content of the PJC sample was 274.30 ± 2.13 mg/100 g (Table 1). The hydrolysis of the glycosidic monomers and the phenolic oligomers found in the PJC releases the different phenolic aglycons and exposes more hydroxyl groups, which react with the total phenolics assay reagent (Folin–Ciocalteu). Our conclusions were consistent with the conclusions of other researchers [1,3,6], while the results previously reported [11] had higher values than our findings. The differences in total phenolic content of PJC between these studies are anticipated to be due to influences such as the types of grenades, climate, cultivars, production, extraction, evaporation, heat performances, and soil [3].

#### 3.1.2. Total Flavonoids

Flavonoids are a subclass of phytochemicals with strong bioactive potential and free radical scavenging capacities, and they positively affect human health [3]. As illustrated in Table 1, the total flavonoid content of the PFC was 82.58 ± 0.75 mg/100 g. These data agree with those of previously published studies [1,3,6], while other results previously reported [11] showed a higher value than our findings. The reasons for the differences in the total flavonoid content of the PJC between these investigations are similar to the reasons for the differences in the total phenolic content.

#### 3.1.3. HPLC Quantification of Flavonoids and Phenolic Acids

Most of the phenolic compounds in the PJC sample were quantified using phenolic acid and flavonoid standards. However, because of the unavailability of standard compounds, additional mixtures with comparable phenolic spectra and chromatographic performance were not identified. The major constituents in PJC were 4-Hydroxy-3-Methoxybenzoic acid 909.72 ± 28.66, followed by epicatechin 532.84 ± 18.70, catechin 310.51 ± 1.07, rutin 287.25 ± 5.42, ferulic acid 150.62 ± 1.91, P-coumaric acid 95.86 ± 3.90, and cinnamic acid 15.13 ± 0.12 mg/100 g (Table 1). In contrast, syringic acid and gallic acid could not be detected. This is the first investigation that provides the phenolic acid profile of PJC. Pomegranate variety, in addition to other factors, including geographical and climatic environments, soil, fertilization, degree of maturity, agronomy methods, extraction evaporation, and heat technique, can influence the phenolic compound structures [3].

### 3.2. Antioxidant Activity

Assuming the inclusion of various antioxidant mechanisms in PJC, the PJC sample’s antioxidant activities were evaluated using several assays. The phenolic compounds’ potential candidates have a significant influence on antioxidant activities. The differences detected between the results presented in the current study and the results in the literature are due to pomegranate variety, the degree of ripeness, growing conditions, fertilizers, soil type, season, geographic origin, storage conditions, diseases, extraction evaporation, and heating methods [3].

#### 3.2.1. Assay for Scavenging DPPH^•^ Free Radicals

**Figure 1:**
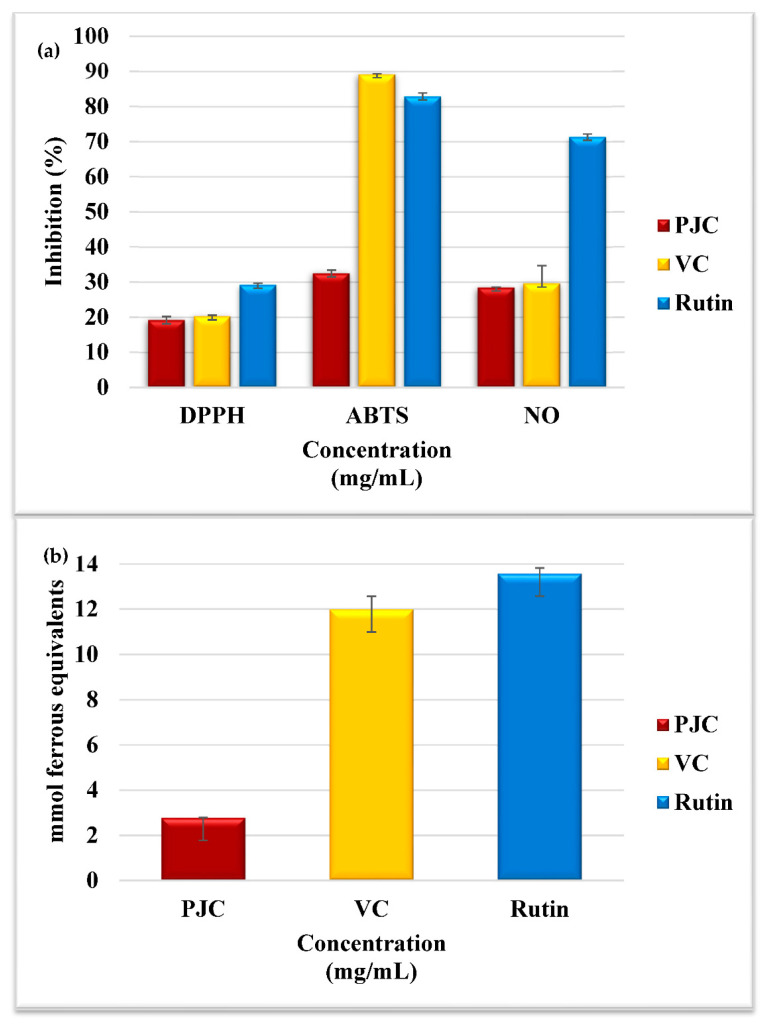
(**a**) Activity of DPPH^•^, ABTS^•^, and NO; (**b**) FRAP assays for PJC, vitamin C, and rutin. Data are expressed as the mean ± standard deviation; *n* = 3.

The free radical DPPH^•^ has been extensively employed in evaluating antioxidant capabilities of eliminating free radicals in vitro. PJC reduced the activity of DPPH^•^, as shown in Figure 1a. The DPPH^•^ scavenging action of PJC was 19.17 ± 1.01%, while the DPPH^•^ scavenging activities for rutin and vitamin C (used as comparison standards and as positive controls) were 29.23 ± 0.41% and 20.18 ± 0.32%, respectively. These results are similar to those previously reported [1,3,6,8,9,11].

#### 3.2.2. Assay for Scavenging ABTS^•^ Free Radicals

This analysis is extensively employed to evaluate the capability of natural antioxidants to remove free radicals [35]. As illustrated in Figure 1a, PJC, VC, and rutin were very effective in eliminating ABTS^•^. The ABTS^•^ scavenging activity of PJC was 32.49 ± 0.94%, while the ABTS^•^ scavenging activities of VC and rutin (comparison standards and as positive controls) were 89.19 ± 0.12% and 82.84 ± 1.04%, respectively.

#### 3.2.3. Assay for Scavenging Nitric Oxide Radicals

Immune cells and cytokines are involved in triggering the overexpression of the enzyme-inducible nitric oxide synthase (iNOS) during bacterial infection. The overexpression of iNOS results in the overproduction of nitric oxide (NO), which is included in the development of inflammation. Hence, scavenging NO may effectively manage inflammatory diseases [31,35]. The % inhibition of NO by PJC, VC, and rutin, as positive controls, were 28.45 ± 0.11, 29.57 ± 5.07, and 71.32 ± 0.85%, respectively, as illustrated in Figure 1a. These results suggest that PJC is capable of inhibiting the damaging effects induced by the overproduction of NO [31,35].

#### 3.2.4. Assay for Measuring the Ferric-Reducing and Antioxidant Power (FRAP)

The FRAP analysis in Figure 1b is based on reducing ferric 2,4,6-tripyridyl-s-triazine complex [Fe (III)-(TPTZ)_2_]^3+^ towards [Fe (II)-(TPTZ)_2_]^2+^ in the presence of antioxidants [31,35]. PJC, VC, and rutin effectively decreased the ferric tripyridyl triazine development. PJC, VC, and rutin, as positive controls, were 2.77 ± 0.01, 11.99 ± 0.58, and 13.58 ± 0.24 mmol ferrous equivalents, respectively. Some bioactive compounds can also chelate metals, thus preventing the oxidative damage caused by the hydroxyl radical.

#### 3.2.5. Total Antioxidant Activity

**Figure 2:**
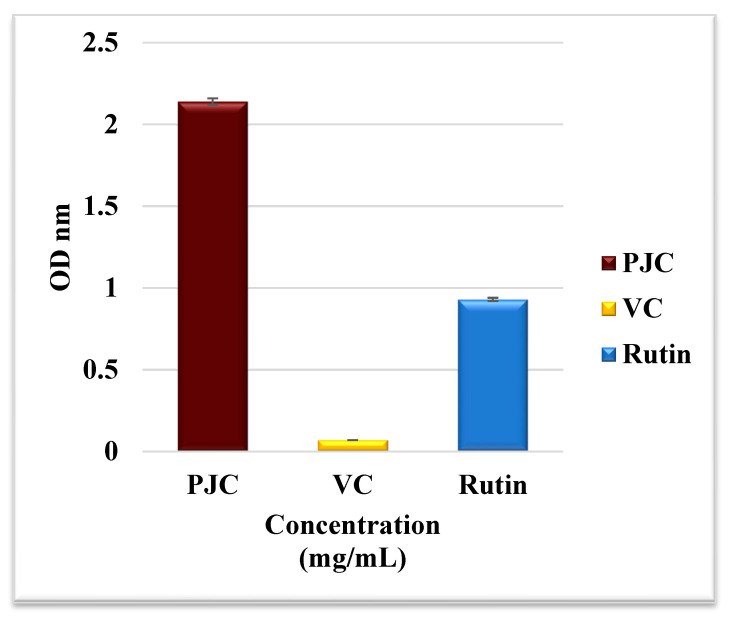
Total antioxidant capacity. Assays for PJC, vitamin C, and rutin. Data are expressed as the mean ± standard deviation; *n* = 3.

The TAC assay revealed the antioxidant capacity of PJC, rutin, and VC, as positive controls, in reducing Mo (VI) to Mo (V) [35]. Figure 2 shows that the concentrations of PJC, VC, and rutin were 2.14 ± 0.02, 0.07 ± 0.00, and 0.93 ± 0.01 OD nM, respectively.

#### 3.2.6. Damage to DNA Caused by Free Radicals

**Figure 3:**
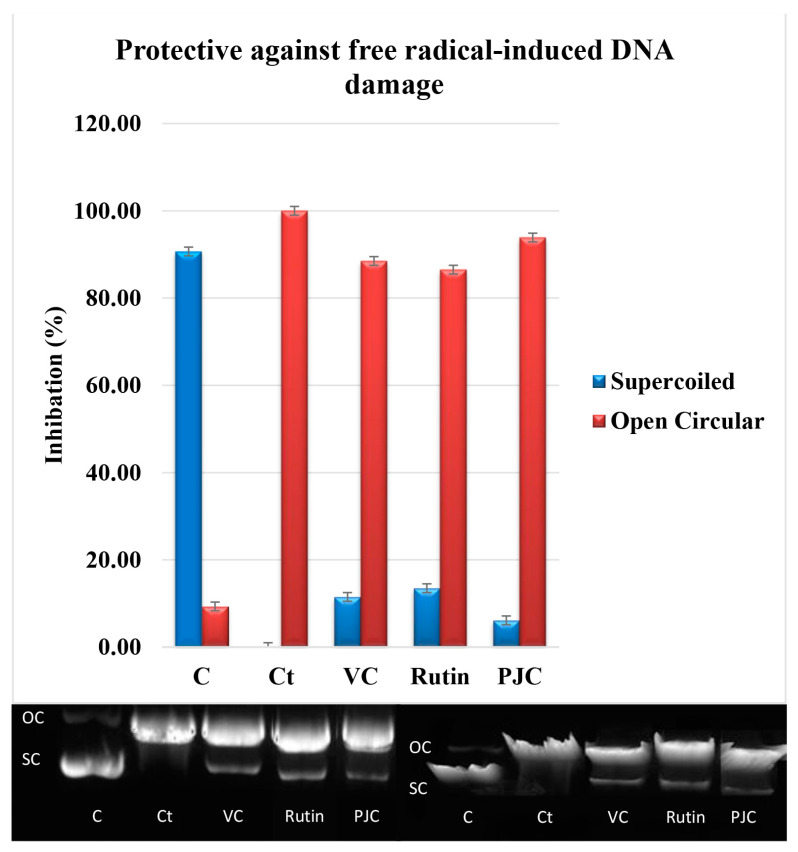
Densitometric analysis, 3Dgel, and normal gel for VC, rutin, and PJC. C: Plasmid; Ct.: plasmid + H_2_O_2_ + UV, plasmid +VC, rutin, and PJC + H_2_O_2_ + UV; OC: open circular; SC: supercoiled. Data are expressed as the mean ± standard deviation; *n* = 3.

This experiment was conducted to establish the effect of PJC on the oxidative damage of plasmid DNA inflicted by OH^•^ produced by UV light plus H_2_O_2_. Three bands resulted from the delapidation of the plasmid DNA: an open circular (OC) band (slow-moving), a natural supercoiled (SC) band (fast-moving), and a linear band (Figure 3). The untreated plasmid showed 90.68 ± 0.07% SC and 9.32 ± 0.07% OC. On the other hand, the plasmid treated with H_2_O_2_, and UV showed 100% OC. The pretreatment with PJC, rutin, or VC guarded against a DNA break, as evidenced by the increase in the SC and a decrease in the OC assembly of DNA. The results of the PJC for the SC DNA and the OC were 2.73 ± 0.26% and 93.87 ± 0.33%, respectively, while those for vitamin C were 11.50 ± 0.63% for the SC DNA and 88.50 ± 0.63% for OC, and those for rutin were 13.50 ± 0.55% for SC DNA and 86.50 ± 0.55% for OC. Rutin, as a positive control, was the most effective compound in preventing damage to pBR322 plasmid DNA, followed by VC, as a positive control, and PJC. This is the first time that PJC has been shown to protect DNA from ionizing effectively. This effect may be due to the ability of PJC to neutralize O2 ^•−^ and (OH^•^).

#### 3.2.7. AAPH-Induced Oxidation of Proteins

**Figure 4:**
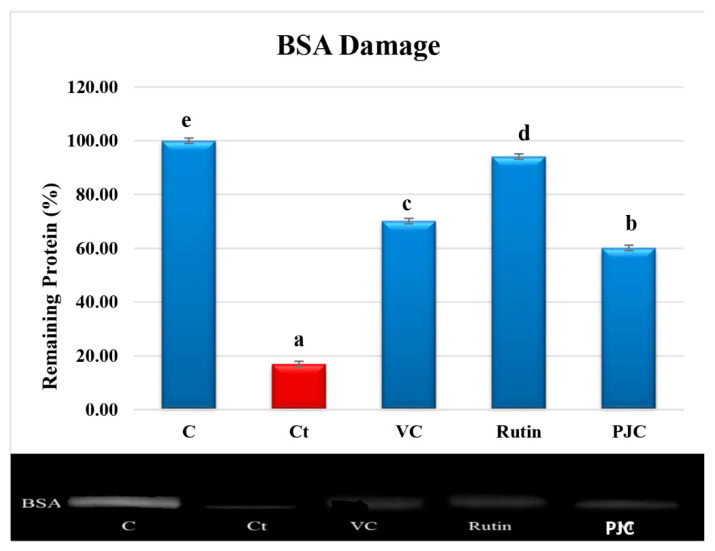
Densitometric analysis and sodium dodecyl sulfate-polyacrylamide gel electrophoresis (SDS-PAGE) of a protective effect against the oxidative damage of BSA for VC, rutin, and PJC. C: BSA; Ct: BSA + AAPH, BSA + AAPH + VC, rutin, or PJC. Data are expressed as the mean ± standard deviation; *n* = 3. Different letters in a column denote significant differences, *p* < 0.05.

The oxidation of proteins by free radicals results in covalent alterations and functional modifications in the protein molecules. The densitometric analyses of the BSA electrophoretic bands following the incubation of BSA with AAPH with PJC, VC, or rutin are shown in Figure 4. The thickness of the control BSA band (lane 1) remained at 100%, and the density of the treated BSA band (lane 2) was 16.97 ± 0.26%, (*p* < 0.05) after incubation with AAPH for 30 min. The treatments with PJC, VC, and rutin (lanes 3–5), as positive controls, stopped the BSA from breaking up. While the breaking up of BSA reduced by 60.14 ± 0.40%, (*p* < 0.05), for PJC, vitamin C decreased by 70.13 ± 0.34%, (*p* < 0.05), and rutin decreased by 94.12 ± 0.20%, (*p* < 0.05). This report shows, for the first time, the protective effect of PJC against oxidative protein damage.

### 3.3. Enzyme Inhibition Activity

#### 3.3.1. Porcine α-Amylase Inhibition Assay

**Figure 5:**
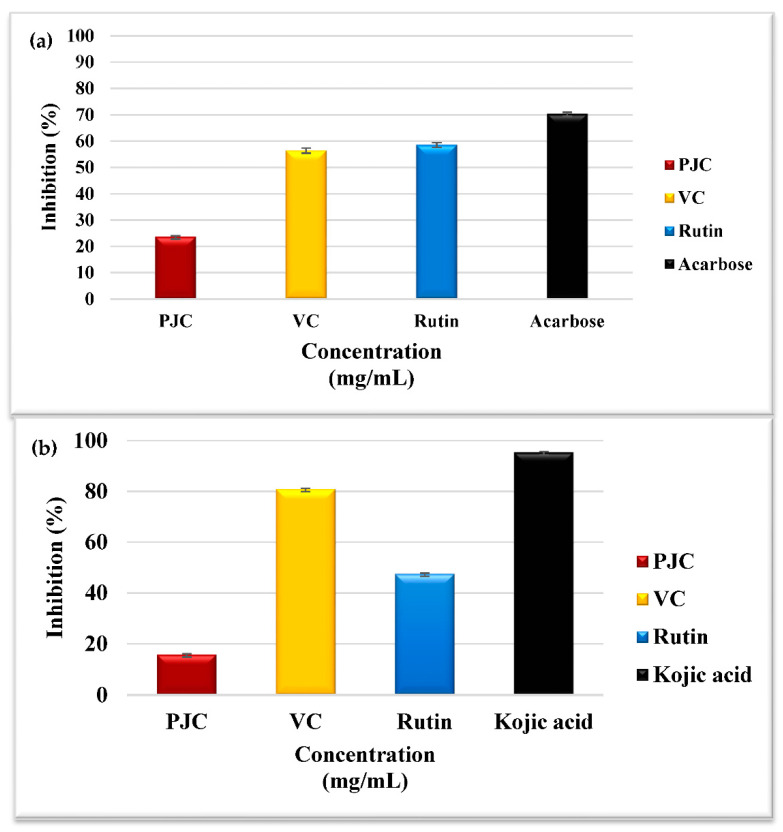
(**a**) Porcine α-amylase inhibition activity; (**b**) Tyrosinase inhibition activity of PJC, VC., rutin, acarbose, and kojic acid. Data are expressed as the mean ± standard deviation; *n* = 3.

Compounds inhibiting amylase activity may reduce postprandial hyperglycemia and potentially treat type 2 diabetes and obesity [35]. The % inhibition of amylase by PJC, VC, acarbose as a positive control, and rutin were 23.75 ± 0.24, 56.51 ± 0.81, 70.41 ± 0.57, and 58.64 ± 0.77%, respectively (Figure 5a). The flavonoids and polyphenols found in PJC could be accountable for stopping amylase, thus proposing a promising treatment for hyperglycemia. The inhibitory action of PJC against amylase is possibly due to hydrogen bonds formed between the proteins in the enzyme’s active site and the hydroxyl groups in polyphenols [35]. Polyphenols have been shown to connect to amylase via hydrogen bonding and to modify its secondary configuration. This blocks the control of the substrate via the enzyme’s active site, leading to its deactivation [35]. Our results align with the only investigation that assessed the inactivation of porcine α-amylase by PJC [3].

#### 3.3.2. Tyrosinase Inhibition Assay

Tyrosinase catalyzes the production of melanin in skin, and hair, and influences the generation of dopaquinone. The excessive production of melanin in the body may contribute to skin cancer and Parkinson’s disease [35]. The percentage of tyrosinase inhibition by PJC, VC, and rutin was 15.81 ± 0.31, 80.98 ± 0.16, and 47.60 ± 0.33%, respectively, and the percent inhibition by kojic acid (a positive control) was 95.46 ± 0.29% (Figure 5b). Our results showed, for the first time, the tyrosinase inhibition activity of PJC. This inhibition might be due to the hydroxyl groups in the polyphenols of PJC. The hydroxyl groups bind to proteins in the enzyme’s active site via hydrogen bonding, resulting in reduced enzyme activity. Furthermore, flavonoids may also inhibit tyrosinase activity by chelating copper ion presence in the enzyme’s active site due to their polyphenolic structure [39,40]. Moreover, Yu et al. [39,40] testified that cinnamic acid and ferulic acid, found in the PJC polyphenols, inhibited tyrosinase activity effectively and are likely to inhibit tyrosinase activity synergistically.

#### 3.3.3. Assay for Inhibiting Acetylcholinesterase

**Figure 6:**
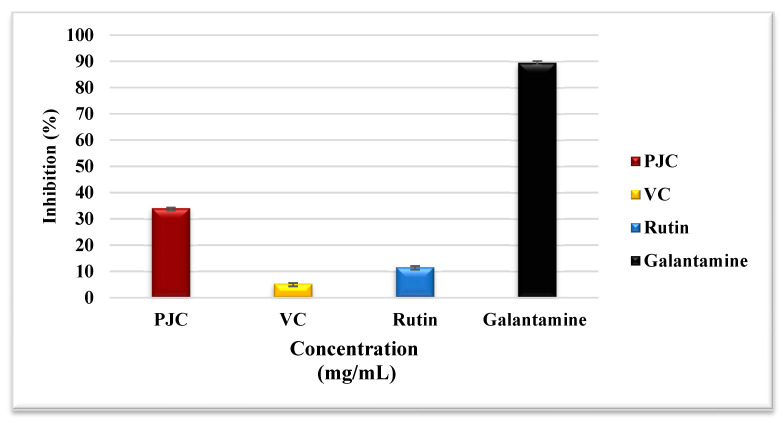
Acetylcholinesterase inhibition activity of PJC, VC, rutin, and galantamine. Data are expressed as the mean ± standard deviation; *n* = 3.

The inhibition of acetylcholinesterase activity attenuates the pathogenesis of Alzheimer’s disease (AD). The medicines used in treating AD have unfavorable side effects and issues associated with toxicity, gastrointestinal disturbances, and toxicity. At the same time, natural enzyme inhibitors may have similar effects on AD treatment with minimal side effects [35]. Figure 6 shows that the percentage of acetylcholinesterase inhibition by PJC, VC, galantamine as a positive control, and rutin were 34.11 ± 0.19, 5.35 ± 0.20, 89.55 ± 0.61, and 11.69 ± 0.37%, respectively. The mechanism(s) of inhibition could involve the reactions of phenolic compounds with the enzyme’s active site, as conferred above for other enzymes. This is the first study that provides evidence of acetylcholinesterase inhibition by PJC.

### 3.4. Antimicrobial Activity 

The excessive misuse of antibiotics assisted the worldwide spread of microbial resistance, which diminishes the potency of the currently marketed antibiotics. Currently, antibiotic resistance is one of the major human health challenges that forces extensive exploration for potent antimicrobial agents with less antibiotic-resistance induction capacity [41]. Medicinal plants and their derivatives are rich sources of novel bioactive compounds with a significant share (about 25%) of the currently applied pharmaceutical compounds [42].

#### 3.4.1. Antimicrobial Assays

**Figure 7:**
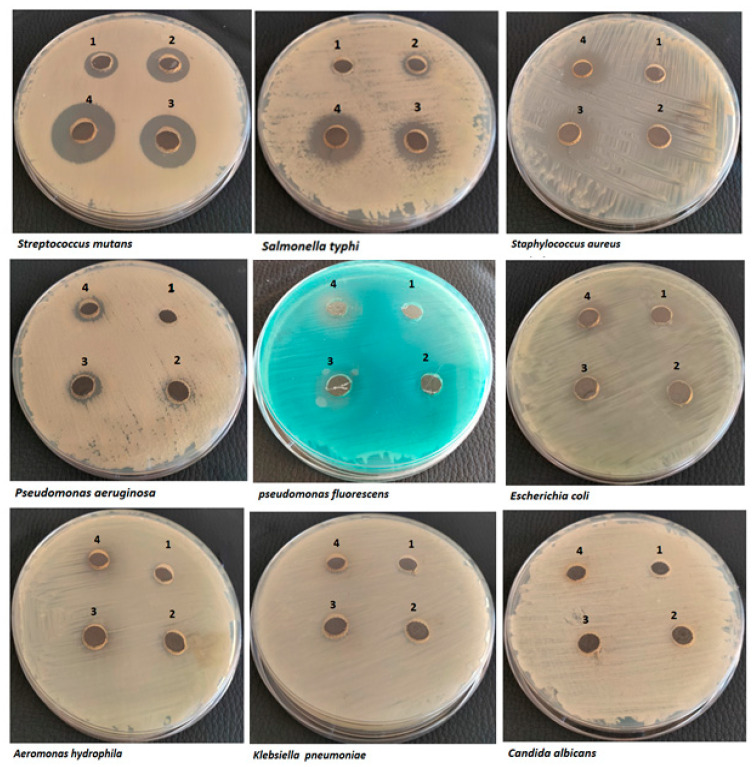
Agar-well diffusion method for antimicrobial activity of PJC at four concentrations against nine different pathogens: 1 (175 µg), 2 (350 µg), 3 (525 µg), and 4 (700 µg). Data are expressed as the mean ± standard deviation; *n* = 3.

The antimicrobial activity of the PJC was assessed against several human bacteria using the agar-diffusion method (Figure 7). The results (Table 2 and Figure 7) indicated concentration-dependent broad-spectrum antibacterial activity of the PJC that varied according to the pathogen type. The results also showed the high potency of PJC toward Gram-positive over that of Gram-negative, which is accordant with other studies on pomegranate peel extracts that attributed the lower sensitivity toward Gram-negative to the outer envelope of the cell membrane [43,44]. The potent PJC activity was against *Streptococcus mutans* with a maximum inhibition zone of 24 ± 1.4 mm at 700 µg. In contrast, the lowermost antibacterial activity was against *Aeromonas hydrophila* (halo-zone of 8 ± 1.76 mm) at the same concentration (Table 2). On the other hand, the results indicated no antibacterial activity against *Klebsiella pneumoniae* at any concentrations, which could be attributed to the outer layer of the polysaccharide (capsule) secreted by the organism that acts as a barrier toward any harmful compounds [45]. In the same regard, the results also indicated no antifungal activity against *Candida albicans.* In the scope of this study, the broad-spectrum antibacterial activity against Gram-positive and Gram-negative bacteria with no antifungal activity indicated that the PJC antibacterial activity could be attributed to the disruption of cell membranes and/or targeting the vital cell process in prokaryotic cells [43].

#### 3.4.2. Minimum Inhibitory Concentration (MIC) Assay

The MIC results corresponded to the disk diffusion as the lowest MIC value was about 175 ± 11.8 µg/mL in *Streptococcus mutans and Salmonella typhi.* In contrast, the highest MIC was 1400 ± 33.5 µg/mL in *Aeromonas hydrophila* (Table 2). The MIC values in this study in the seven tested organisms are much lower than those reported in other studies using pomegranate peel extracts, which asserted the potency of PJC as a potential antibacterial source [43,44].

### 3.5. Anticancer Activity

**Figure 8:**
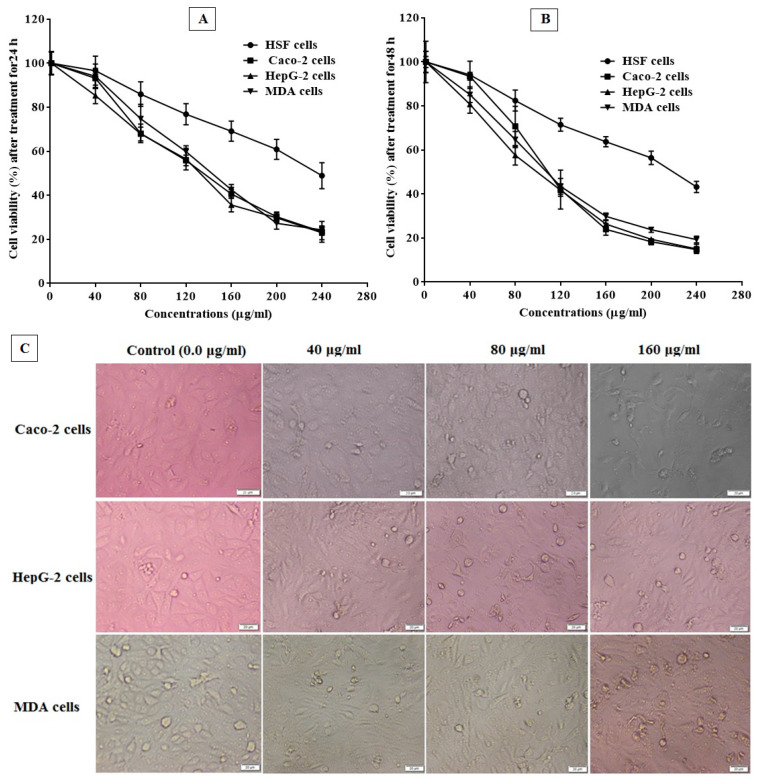
Cytotoxicity profile of PJC against both cancer and normal cell lines. Regular (HSF) cells and cancer (Caco-2, HepG-2, and MDA) cells were exposed to PJC at various dilutions (40–240 μg/mL) for 24 h (**A**) and 48 h (**B**). The experiment of the cell viability was evaluated relative to untreated cells using the MTT method. (**C**) Morphological changes in cancer cells as visualized under the inverted phase-contrast microscope after treatment for 48 h with PJC at different ratios of 40.0, 80, and 160 μg/mL as compared to untreated control cells (0.0 μg/mL). Data are expressed as the mean ± standard deviation; *n* = 3.

The cytotoxic effect of PJC was evaluated for its safety on normal cells and its anticancer activity against cancer cells using the MTT assay. We investigated the sensitivity of HSF (normal), MDA (breast carcinoma), HepG-2 (hepatoma), and Caco-2 (colon carcinoma) cell lines to PJC treatment at different doses, which indicates its anticancer selectivity. The results in Table 3 show that the values of EC_100_ and IC_50_ for PJC were 6.4–10.1 times higher on the tested normal cells than on cancer cells after treating them for 24 and 48 h. Table 3 indicates that PJC had anticancer action against Caco-2, HepG-2, and MDA cells at estimated IC_50_ values of 129.9 ± 1.34, 164.4 ± 1.61, and 141.6 ± 1.35 μg/mL, respectively, with SI values of 8.09 ± 0.23, 6.39 ± 0.18, and 7.43 ± 0.21, respectively, after 24 h of conduct. However, the IC_50_ values were estimated to be 100.5 ± 5.13, 106.3 ± 9.68, and 101.2 ± 6.86 μg/mL plus SI values of 10.09 ± 0.15, 9.55 ± 0.14, and 10.03 ± 0.15 for Caco-2, HepG-2, and MDA cells, respectively, after a 48 h treatment. In addition, Figure 8A,B indicate that PJC exerted a cytotoxic effect against cancer cells in a dose-dependent manner with a significant increase in its selectivity and safety against normal HSF cells. The results are in accordance with [46], which reported the ability of pomegranate extracts to inhibit the growth progression of the colon (Colo205) and pancreatic (Suit-2) cancer cells in a membrane model (chick chorioallantoic membrane). In the same regard, pomegranate juice was reported to boost the anticancer effect upon combing with cisplatin (at low doses) against the A549 cell line of human lung cancer [47]. Furthermore, Figure 8 C presents the relative morphological differences in treated Caco-2, HepG-2, and MDA cells regarding treatment among PJC (40, 80, and 160 μg/mL) for 48 h. Thus, all photomicrographs reveal that the morphology of all treated cancer cells was dose-dependently altered with a pronounced induction of cell destruction. These morphological changes include cell shrinkage, blabbing, and nuclear condensation (Figure 8C).

**Figure 9:**
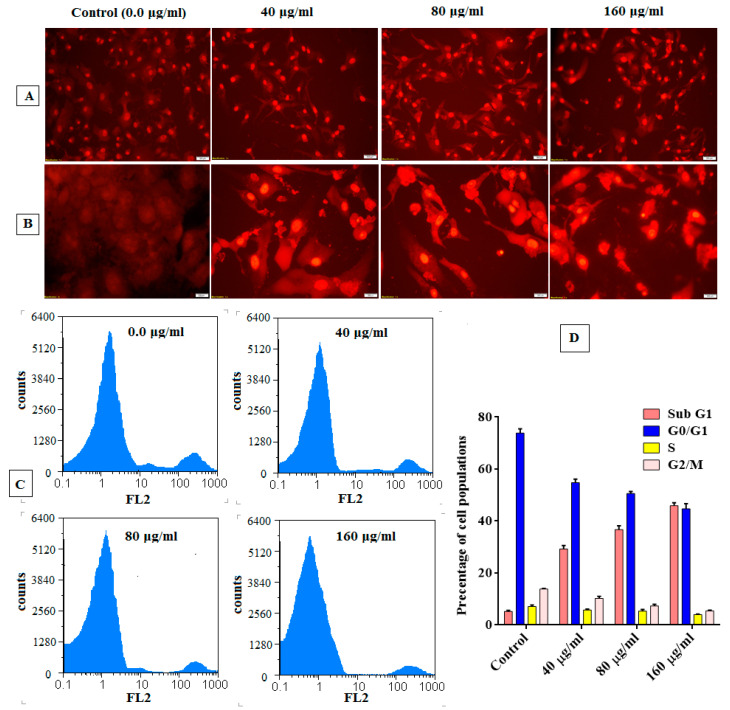
Apoptotic effect of PJC against HepG-2 cells. (**A**) Fluorescence nuclear staining using PI dye. (**B**) Fluorescence images of nuclear staining using ethidium bromide-acridine orange of HepG-2 cells. (**C**) Original flow charts of cell cycle analysis of treated-HepG-2 cells. (**D**) Quantitative distribution of the treated HepG-2 cells in different phases of the cell cycle. Untreated cells were included as a control reference. HepG-2 cells were exposed to PJC at concentrations of 40, 80, and 160 μg/mL for 48 h.

On the other hand, the nuclear changes captured in the HepG-2 cells treated with PJC confirm that the anticancer activities of PJC were exerted by the apoptotic mechanism (Figure 9A,B). The apoptotic effect was induced at the early and late stages in the treated HepG-2 cells, as made evident by the AO/EB fluorescent nuclear staining. The membrane integrity of these cells has significantly been lost, as evidenced by the release of yellow and orange fluorescence compared to green fluorescence in negative control cells. The results accord with the reported potential of permanganate ingredients to induce and modulate the signaling, transcriptional, and apoptotic factors that control the cell cycle in malignant cells [48]. Furthermore, the detected apoptotic effect of PJC was enhanced dose-dependently, as indicated in Figure 9B, in which the nuclei of PI-stained HepG-2 cells became more condensed with chromatin fragmentation with the increase in PJC concentration compared to untreated HepG-2 cells (control). The dose-dependency of pomegranate fruit extract to inhibit bladder cancer development was also previously reported [49] and attributed to its anti-inflammatory and antioxidant potentials. Moreover, the cell cycle division profile of the PJC-treated HepG-2 cells was analyzed to confirm its apoptotic mechanism, which showed an enhancement in cell cycle arrest (Figure 9C). The growth distribution of cell population in both G0/G1 and G2/M (main checkpoints phases) was changed with a noticeable increase in fractions of the sub-G1phase after increasing the treatment dose (40, 80, and 160 μg/mL). Nevertheless, the production (S) phase is reduced dose-dependently, as shown in Figure 9D. These results demonstrate the capability of PJC to stimulate the cell cycle arrest of the treated HepG-2 cells compared to untreated reference cells and induce the apoptotic effect pathway as the primary mechanism of anticancer activity.

**Figure 10:**
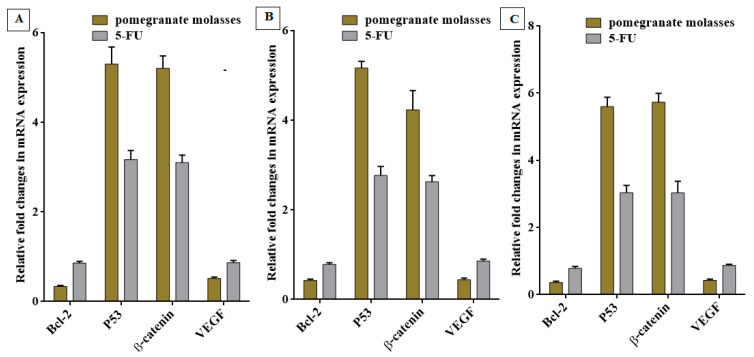
Estimation of changes in expression levels of four key genes, including B-cell lymphoma 2 (Bcl-2), tumor protein (p53), Catenin beta-1 (β-catenin), and Vascular Endothelial Growth Factor (VEGF), relative to untreated control cells via qPCR. Angiogenesis-related genes are estimated in (**A**) colon carcinoma (Caco-2), (**B**) hepatoma (HepG-2), and (**C**) breast carcinoma (MDA) cells exposed to PJC compared with fluoropyrimidine 5-fluorouracil (5 FU) at IC50 values for 48 h. All values are stated as mean ± SEM and stand for the average values from *n* = 3.

Furthermore, Figure 10A–C reveals that PJC can down-regulate both oncogenes (BCl-2 and VEGF) and up-regulated p53 and β-catenin genes in the treated MDA, Caco-2, and HepG-2 cell lines. PJC (IC_50_ dose) has a potent activity to suppress the expressions of Bcl-2 and VEGF genes and increase p53 and β-catenin expression stages by 5–6-fold more than control cells and 1.5–2-fold more than the 5-FU-treated cells.

### 3.6. Correlation Analyses

The results show the correlation coefficients between total phenolic (TP) content, total flavonoid (TF) content, various phytochemical compounds (hydroxy3, catechin, epicatechin, coumaric, ferulic, rutin, and cinnamic acid), and antioxidant and enzyme inhibitory activities (DPPH, ABTS, FRAP, TAC, NO, tyrosinase, acetylcholinesterase, and amylase). The correlation coefficients ranged from −1 to 1, with positive values indicating a positive correlation and negative values indicating a negative correlation between the two variables. The closer the value is to 1 or −1, the stronger the correlation between the variables.

**Figure 11:**
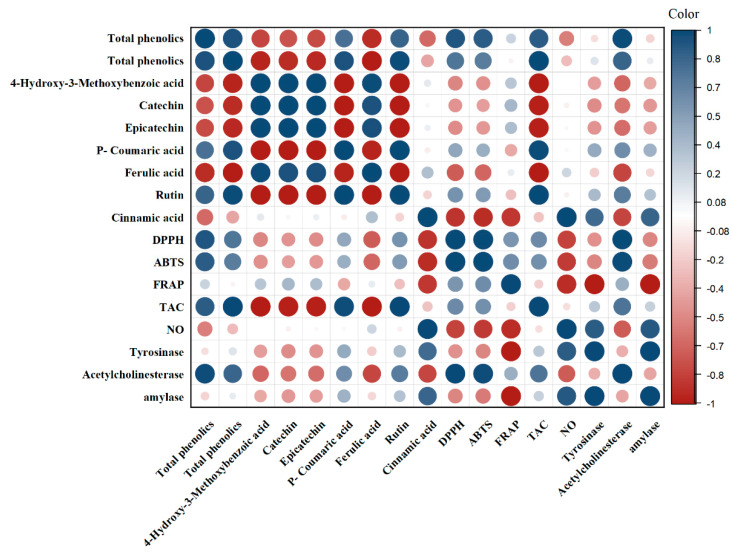
A color diagram was used to evaluate the correlation heatmap among the contents of TP, TF, phenolic compounds, and antioxidant activity in PJC.

The results in Figure 11 indicate that TP and TF have a strong positive correlation with each other (r = 0.952), which is expected as they are both measures of the phenolic content of the samples. TP and TF also showed a strong positive correlation with most of the individual phytochemical compounds, except for Hydroxy3, which showed a negative correlation with both TP and TF. This could be due to the fact that Hydroxy3 is a type of flavonoid and may have a different relationship with TP and TF compared to other types of phytochemicals. The antioxidant activities (DPPH, ABTS, FRAP, and TAC) showed a strong positive correlation with most of the phytochemical compounds, indicating that these compounds contribute significantly to the antioxidant potential of the samples. However, the correlation coefficients between the phytochemicals and the enzyme inhibitory activities (tyrosinase, acetylcholinesterase, and amylase) were generally weaker, indicating that other factors may also contribute to these activities.

In conclusion, the results suggest that the phenolic content and various individual phytochemicals in the samples are strongly correlated with their antioxidant activities, but their relationship with enzyme inhibitory activities is weaker. These findings may have implications for the development of functional foods and nutraceuticals with specific health benefits.

## 4. Conclusions

This study presents an innovative investigation of the polyphenolic profile of PJC; its effects on DNA and BSA oxidative damage; and the inhibition of some clinical enzymes, cancer cells, and pathogenic bacteria in vitro. The results from the current study show, for the first time, that the main polyphenols in PJC are 4-Hydroxy-3-Methoxybenzoate, epicatechin, catechin, rutin, ferulic acid, P-coumarate, and cinnamic acid. The results also show that PJC effectively inhibits DNA and BSA damage mediated by free radicals, indicating the substantial potential of PJC to defend against the oxidative injury of these vital biomolecules. Furthermore, PJC also inhibited the activities of acetylcholinesterase and tyrosinase, which have been implicated in the pathogenesis of several disease states. This study also supports the function of PJC in triggering the death of breast, hepatic, and colon cancer cells via apoptosis and by inhibiting pathogenic bacteria, such as *Streptococcus mutans* and *Aeromonas hydrophila*. These findings attempt to create a better recognition of the health-promoting properties of the bioactive compounds found in PJC and further support the use of PJC in nutraceuticals and functional food products.

## Figures and Tables

**Table 1:** The bioactive compounds present in PJC.

Bioactive Compounds mg/100 g PJC										
TP	TF	Gallic Acid	4-Hydroxy-3- Methoxybenzoic Acid	Catechin	Syringic Acid	Epicatechin	P-Coumaric Acid	Ferulic Acid	Rutin	Cinnamic Acid
274.30 ± 2.13	82.58 ± 0.75	nd	909.72 ± 28.66	310.51 ± 1.07	Nd	532.84 ± 18.70	95.86 ± 3.90	150.62 ± 1.91	287.25 ± 5.42	15.13 ± 0.12

Nd: Not detected; TF: total flavonoids, (mg RE/100g); TP: total phenolics, (mg GAE/100 g). All values are expressed as mean ± SD; *n* = 3.

**Table 2:** Antimicrobial activity of PJC against different pathogenic organisms represented by halo-zone diameter (mm) and MIC values (µg/mL).

Pathogen	Halo-Zone Diameter (mm)				MIC (µg/mL)
	175 µg	350 µg	525 µg	700 µg	
*Streptococcus mutans*	11 ± 1.20	16 ± 0.97	20 ± 1.40	24 ± 1.40	175 ± 11.80
*Salmonella typhi*	0.0 ± 0.0	7 ± 1.57	13 ± 0.77	20 ± 0.97	175 ± 12.60
*Staphylococcus aureus*	0.0 ± 0.0	8 ± 0.17	18 ± 1.20	19 ± 0.78	350 ± 14.55
*Pseudomonas aeruginosa*	0.0 ± 0.0	7 ± 1.78	11 ± 0.97	11.3 ± 1.40	350 ± 11.30
*Pseudomonas fluorescens*	0.0 ± 0.0	00 ± 0.0	13.5 ± 0.78	16.8 ± 1.30	350 ± 9.11
*Escherichia coli*	0.0 ± 0.0	11 ± 1.78	11.5 ± 0.77	14.5 ± 1.78	700 ± 22.50
*Aeromonas hydrophila*	0.0 ± 0.0	0.0 ± 0.0	7 ± 1.98	8 ± 1.76	1400 ± 33.5
*Klebsiella pneumoniae*	0.0 ± 0.0	0.0 ± 0.0	0.0 ± 0.0	0.0 ± 0.0	-
*Candida albicans*	0.0 ± 0.0	0.0 ± 0.0	0.0 ± 0.0	0.0 ± 0.0	-

Data are expressed as the mean ± standard deviation; *n* = 3.

**Table 3:** EC_100_, IC_50_ (μg/mL), and SI values of PJC against HSF, HepG-2, Caco-2, and MDA cell lines following 24 and 48 h treatment.

Cells	24 h			48 h		
	EC_100_	IC_50_	SI	EC_100_	IC_50_	SI
HSF	33.28 ± 0.95	1052 ± 29.87	-	32.11 ± 0.48	1015 ± 15.14	-
Caco-2	4.11 ± 0.04	129.9 ± 1.34	8.09 ± 0.23	3.18 ± 0.16	100.5 ± 5.13	10.09 ± 0.15
HepG-2	5.20 ± 0.05	164.4 ± 1.61	6.39 ± 0.18	3.36 ± 0.31	106.3 ± 9.68	9.55 ± 0.14
MDA	4.48 ± 0.04	141.6 ± 1.35	7.43 ± 0.21	3.20 ± 0.22	101.2 ± 6.86	10.03 ± 0.15

Data are expressed as the mean ± standard deviation; *n* = 3.

## Data Availability

Not applicable.
